# Domain-wall dark pulse generation with SMF-GIMF-SMF structure as artificial saturable absorber

**DOI:** 10.1038/s41598-024-52640-0

**Published:** 2024-01-25

**Authors:** Yu Chen, Tiu Zian Cheak, Tan Sin Jin, G. Vinitha, Kaharudin Dimyati, Sulaiman Wadi Harun

**Affiliations:** 1 Chongqing Vocational Institute of Engineering, No. 1, North and South Avenue, Binjiang New City, Jiangjin District, Chongqing, 402260 China; 2 Photonics Engineering Laboratory, Department of Electrical Engineering, Faculty of Engineering, University of Malaya, 50603 Kuala Lumpur, Malaysia; 3 Faculty of Engineering and Quantity Surveying, INTI International University, 71800 Nilai, Negeri Sembilan Malaysia; 4 Department of Physics, School of Advanced Sciences, VIT, Chennai, Tamil Nadu 600127 India; 5 Faculty of Engineering, UOW Malaysia KDU University College, 40150 Shah Alam, Selangor Malaysia

**Keywords:** Electrical and electronic engineering, Optics and photonics

## Abstract

We experimentally demonstrated the generation of domain-wall dark pulse in an Erbium-doped fiber laser using the combination of a 10 cm graded index multimode fiber sandwiched by single mode fibers as artificial saturable absorber. The interaction of phase difference in grade index multimode fiber allowed the stable dual-wavelength oscillation in the cavity. The dual-wavelength centered at 1567.2 nm and 1569.4 nm produces the topological defect in temporal domain and achieved a dark pulse formation with repetition rate of 21.5 MHz. The highest average pulse energy is calculated as 769.6 pJ with pulse width of 5 ns. Throughout the operating pump power range, the average pulse energy and output power increase linearly, with R^2^ of 0.9999 and achieved the laser efficiency of 9.33%. From the measurement in frequency domain, the signal-to-noise ratio is measured as 49 dB. As compared to reported DW dark pulse works, the proposed structure only required a short length of multimode fiber, which allowed the domain-wall dark pulse to achieve higher pulse repetition rate. The venture of domain wall dark pulse is potentially to pave the foundation toward sustainable industrial growth.

## Introduction

In the year 2023, the contribution of Pierre Agostine, Ferenc Krausz and Anne L’Huillier in generating attosecond pulses of light that can give a snapshot of changes within atoms has been awarded Nobel Prize in Physics^1^. The research in ultrafast phenomena is one of the highlighted directions in photonics engineering. Ultrafast laser is important to accelerate the current technologies as well as to venture the future technologies. The silhouette of ultrafast laser appears in most of the advanced precision micromachining and surface structuring of emerging materials^2^, particularly in semiconductor and electronics industries for cutting and drilling microstructures^3^. For precise surgery applications, ultrafast lasers have proven its exceptional features in eye surgery dental treatments and microsurgery. Their ability to remove tissue with minimal thermal damage makes them suitable for delicate surgical procedures^4^. Toward the future technologies, ultrafast lasers driving the advancement in attosecond science, which creates new possibilities for understanding and manipulating photons and electrons at the atomic and subatomic levels^5^.

In fiber laser system, ultrafast phenomena are being all-rounded investigated through different types of solitons. In general, the solitons can differentiate into bright solitons^6^ and dark solitons^7^. Bright and dark solitons are two solitary wave solutions that can be elaborated by Nonlinear Schrödinger Equation (NLSE)^8^. Bright solitons are localized, solitary wave solution in NLSE, it is typically expressed as hyperbolic secant function, with localized peak under a background of continuous wave (CW)^9^. On the other hand, as another side of NLSE solution, dark soliton is typically expressed as hyperbolic tangent function, with a localized dip under a background of CW^10^. To date, bright pulses have been widely used in most industries, however, dark pulses exhibit some unique properties that being described as “a solution looking for a problem”. Dark pulse exhibits better immunity to propagation loss and noise suppression^11^. Moreover, unlike the high pulse peak power of bright pulse that easily induces unwanted nonlinear effects, the absence of energy in CW background allows the dark pulse to propagate with very minimum pulse degrading^12^. These properties are advantageous to maintain the pulse characteristics that are essential to achieve high speed long-haul telecommunication^13^.

Dark pulses, or dark solitons are well-elaborated by NLSE, however, the physical development of dark pulse in fiber laser system is not as advanced as the development of bright pulse fiber laser. Based on the equation, dark pulses can be induced in normal dispersion fiber laser cavity^14^, called as NLSE dark pulse. Alternatively, when the higher order of non-Kerr nonlinearities dominated in the fiber laser cavity, Cubic-Quintic NLSE (CQNLSE) dark pulse was achievable in this condition^15^. These requirements increase the difficulties of generating dark pulse in fiber laser system.

Apart from NLSE dark pulse and CQNLSE dark pulse, there is another type of dark pulse formation called domain-wall (DW) dark pulse^16,17^. The fundamental elaboration of DW dark pulse is the interaction among two or more lases at different wavelength to induce the topological defects in temporal domain. As compared to NLSE dark pulse or CQNLSE dark pulse, DW dark pulse not constrained by dispersion or high order nonlinearities, which simplified the design of dark pulse generation in fiber laser system^12^. As DW dark pulse required two or more lases to interact, the fiber laser cavity usually consists of interferometer mechanism to achieve stable multiple wavelength oscillation, including tapered fiber and nonlinear polarization rotation (NPR)^18^. However, tapered fiber is fragile to incorporate in fiber laser system^19^, whereas NPR required long length of nonlinear medium to achieve sufficient rotation.

In this work, we experimentally demonstrated the generation of DW dark pulse in an Erbium-doped fiber laser (EDFL) using single-multi-single (SMS) mode fiber as interferometer^20^ and artificial saturable absorber^21,22^. The SMS fiber induces phase difference to the oscillating light and produces stable dual-wavelength. Furthermore, with the incorporation of polarization controller (PC) with SMS fiber, it can function as a polarizer to filter low intensity mode and to aid the dark pulse formation^23^. As compared to reported DW dark pulse works or loop mirror type of artificial saturable absorber^24^, the SMS fiber only required a short length of multimode fiber, which allowed the DW dark pulse to achieve higher frequency.

## Experiment setup

**Figure 1:**
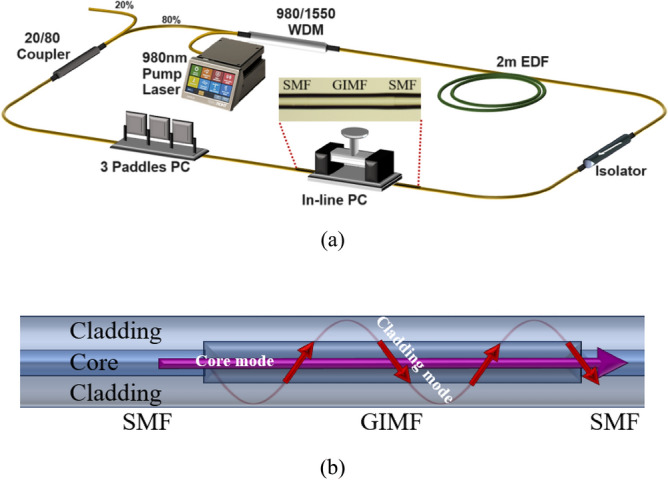
(**a**) Experimental setup of the domain-walled dark pulse EDFL with SMF-GIMF-SMF as artificial saturable absorber. (**b**) Light propagation in SMF-GIMF-SMS structure.

The experimental setup of the mode-locked Erbium-doped fiber laser with SMS structure as SA is shown in Fig. 1. The fiber laser system is pumped by laser diode (Oclaro LC96A74P-20R) with a center wavelength of 974 nm. The pump laser and oscillation light are coupled by 980/1550 nm wavelength division multiplexer (WDM). The common port of the WDM is connected to a 2 m long Erbium-doped fiber (EDF, DF1100 Fibercore), which functions as gain medium. The laser output from the EDF was spliced to an isolator to ensure a unidirectional light oscillation inside the ring cavity. The 10 cm graded index multimode fiber (GIMF) (YOFC, OM4) is sandwiched between single mode fibers (SMFs) to form a SMF-GIMF-SMF structure. When the light propagates through the SMF-GIMF-SMS structure, the core mode experiences refraction at the SMF-GIMF interface. As the GIMF supports multimode, the cladding mode could be formed on top of core mode. The difference in the effective refractive of two propagating modes in the GIMF results in a significant phase difference. Thus, an interference pattern is produced when both core and cladding mode recombine at the end-point of second SMF-GIMF interface as shown in Fig. 1b. The GIMF is incorporated inside an in-line polarization controller (PC) (OEABT, NPC-F). By adjusting the compression upon the GIMF, the phase of light oscillation could alter accordingly. A 3 paddles PC is connected next to the in-line PC. By proper adjustment of the 3 paddles PC, it can function as a polarizer to filter low intensity mode and to aid the dark pulse formation. The ring cavity is completed by a 20/80 coupler, in which 80% of oscillating light is looped in the ring cavity, and 20% of oscillating light is tapped out from the ring cavity for further analysis. The optical spectrum is analyzed by optical spectrum analyzer (OSA) (Anritsu, MS9710C) with a resolution of 0.02 nm. A high-speed photodetector (ET-3500F) attached to digital oscilloscope (GW Instek, GDS-3352) to monitor the real time signal in temporal domain. The stability of the dark pulse is checked in frequency domain, using RF spectrum analyzer (Anritsu, MS2830A). The total length of the ring cavity is around 9.5 m, with net dispersion of ~ -0.118 ps^2^.

## Results and discussion

**Figure 2:**
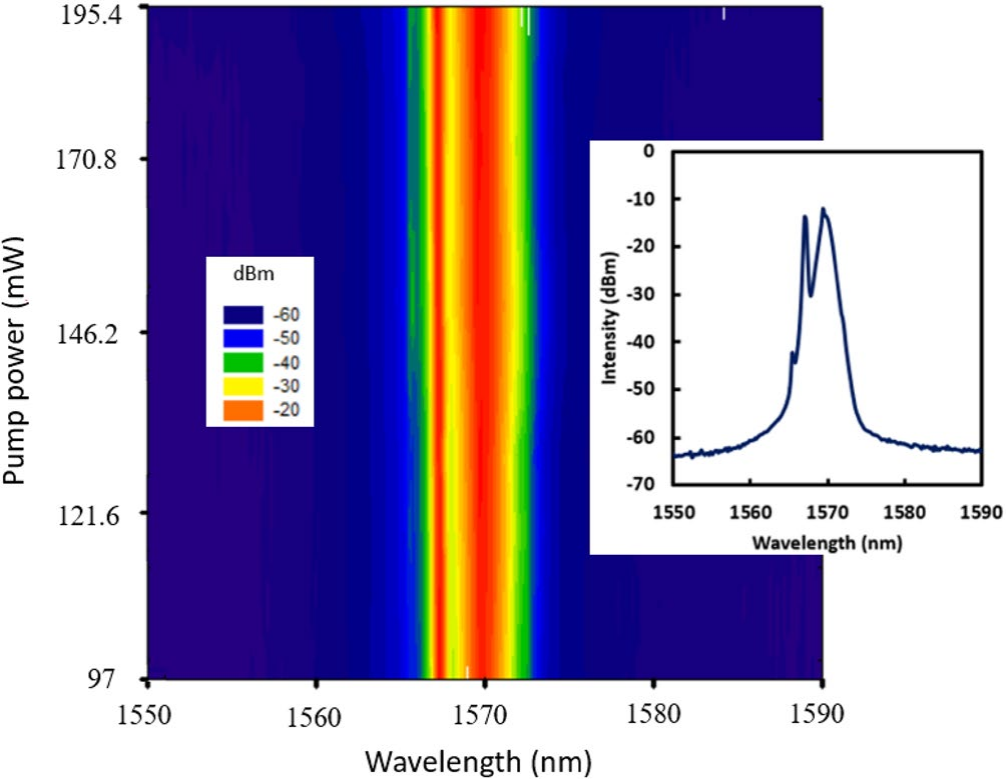
Wavelength domain of DW dark pulse throughout the operating pump power range. Inset is the optical spectrum at pump power of 195.4 mW.

The formation of DW dark pulse is relied on interaction of two or more lasing centered at different wavelengths to induce the topological defects in temporal domain. Mathematically, DW exhibits as kinks, by applying Bӓcklund transformation to the NLSE, and the single-kink soliton is described by^25^

1 $$A\left( {z,t} \right) = 2\sigma \tanh \left[ {\sqrt 2 \sigma_{0} \left( {t - t_{0} } \right) + 4\xi_{0} z} \right]exp\left\{ {i\left[ { - 2\xi_{0} t - 4\left( {\sigma_{0}^{2} + \xi_{0}^{2} } \right)z - \theta_{0} } \right]} \right\}$$

where $\sigma_{0}$ and $\xi_{0}$ are two real parameters, and the real constants $\theta_{0}$, $t_{0}$ fix the location of the traveling wave at the initial time. From above equation, the formation of DW dark pulse is independent from dispersion condition, and it can be formed in either normal or anomalous dispersion fiber laser cavity. In the proposed cavity setup, the SMF-GIMF-SMF served as interferometer to produce stable dual-wavelength. Furthermore, by properly adjusting the 3 paddles PC, it can function as a polarizer to filter low intensity mode and to aid the dark pulse formation. On the whole, the combination of SMF-GIMF-SMF and PCs are forming an artificial saturable absorber to induce DW dark pulse in the cavity. The DW dark pulse is self-started at pump power of 97.0 mW, and stably observed until maximum pump power of 195.4 mW. The dual-wavelength is clearly observed as shown in Fig. 2. The interaction of dual peak wavelength centered at 1567.2 nm and 1569.4 nm produces the topological defect in temporal domain^12^. As presented in 2D contour view, the dual-wavelength are stably oscillating in the ring cavity throughout the pump power range from 97.0 to 195.4 mW.

**Figure 3:**
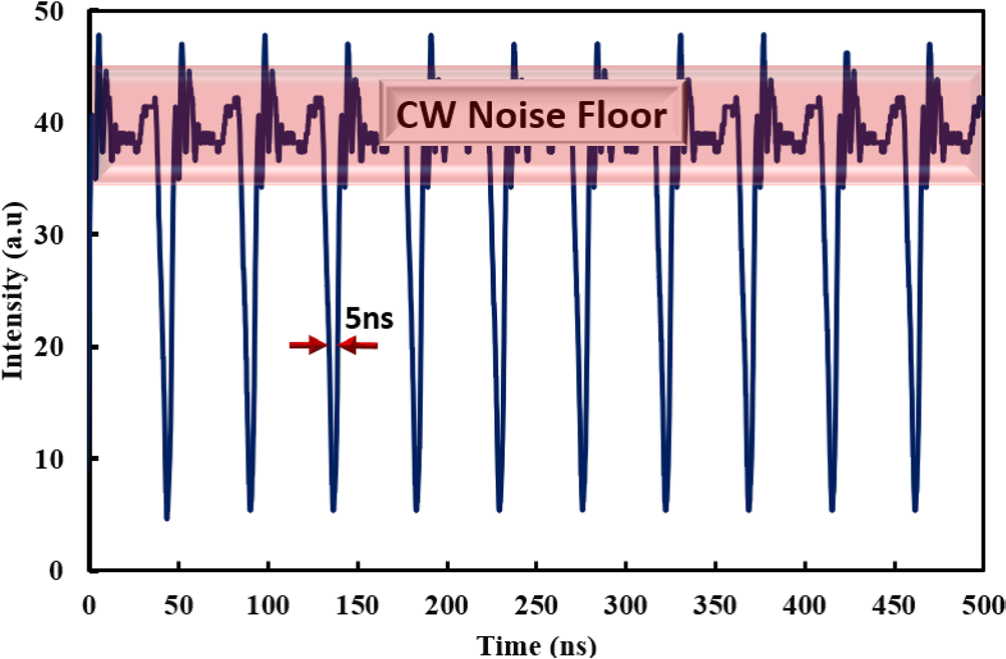
Temporal domain of DW dark pulse at pump power of 195.4 mW.

The DW dark pulse is tangibly visualized in temporal domain as shown in Fig. 3. When the dual lasing oscillates simultaneously cavity, it leads to the emergence of topological defects in temporal domain and forms a narrow intensity dip under the strong continuous wave (CW) emission background. The pulse repetition rate is recorded as 21.5 MHz, which is tallied with the fundamental frequency of the 9.5 m cavity length. The full width half maximum (FWHM) of the dip is measured as 5 ns. Compared with artificial saturable absorbers, emerging materials as saturable absorbers are more favorable to achieve higher repetition rate^26,27^. However, artificial saturable absorber does not require chemical fabrication process, and can be done with the formation of optical components.

**Figure 4:**
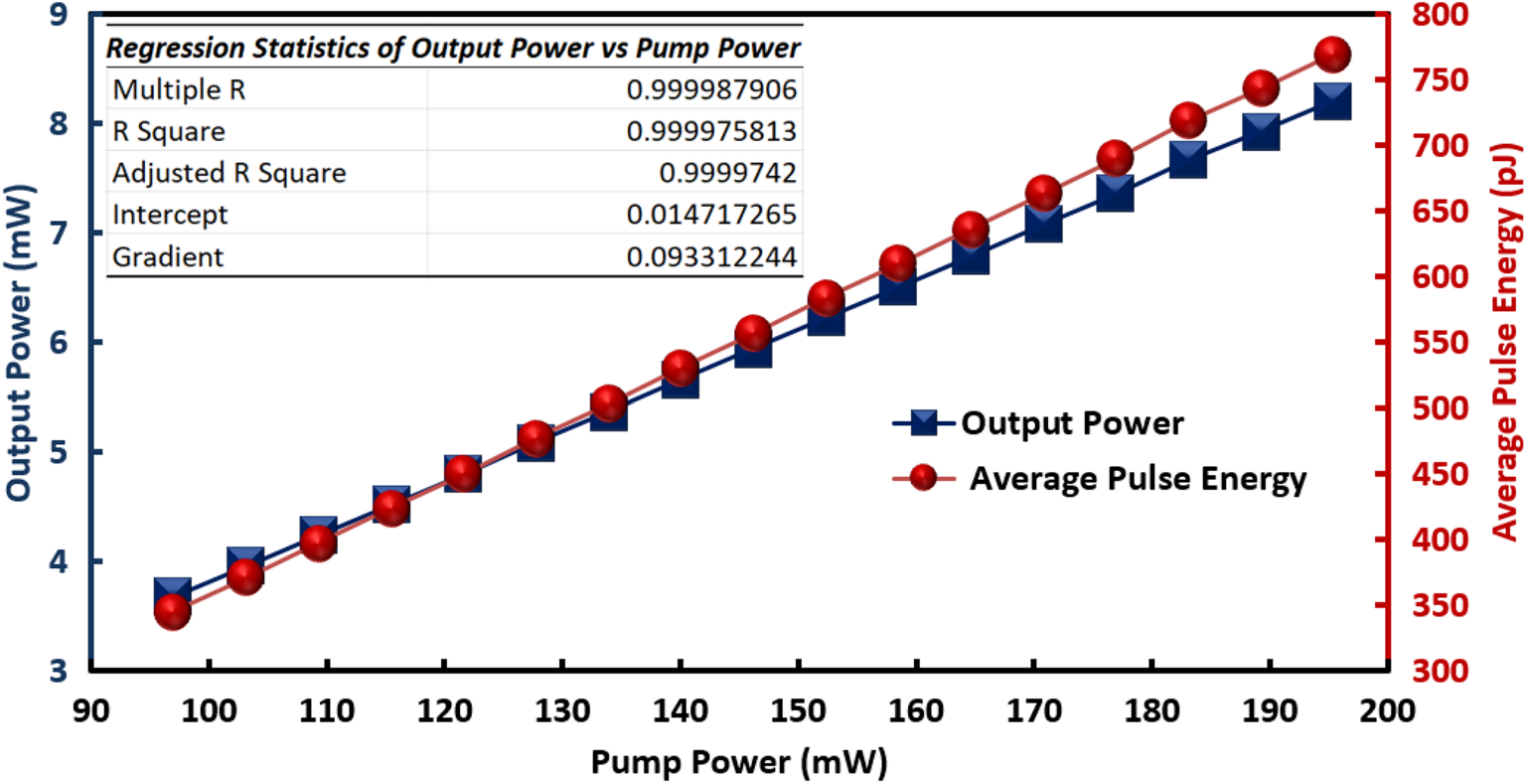
Output power and average pulse energy against pump power.

The DW dark pulse EDFL performance is further analyzed as shown in Fig. 4. Both output power and average pulse energy exhibited a linear increasing trend against pump power. The output power increased from 7.4 to 16.6 mW as pump power increased from 97.0 to 195.4 mW. Besides, the average pulse energy increased from 345.0 to 769.6 pJ within the same pump power range. From the regression statistics of output power against pump power, the laser performance is highly linear with R^2^ of 0.9999 and the laser efficiency is reported as 9.33%.

**Figure 5:**
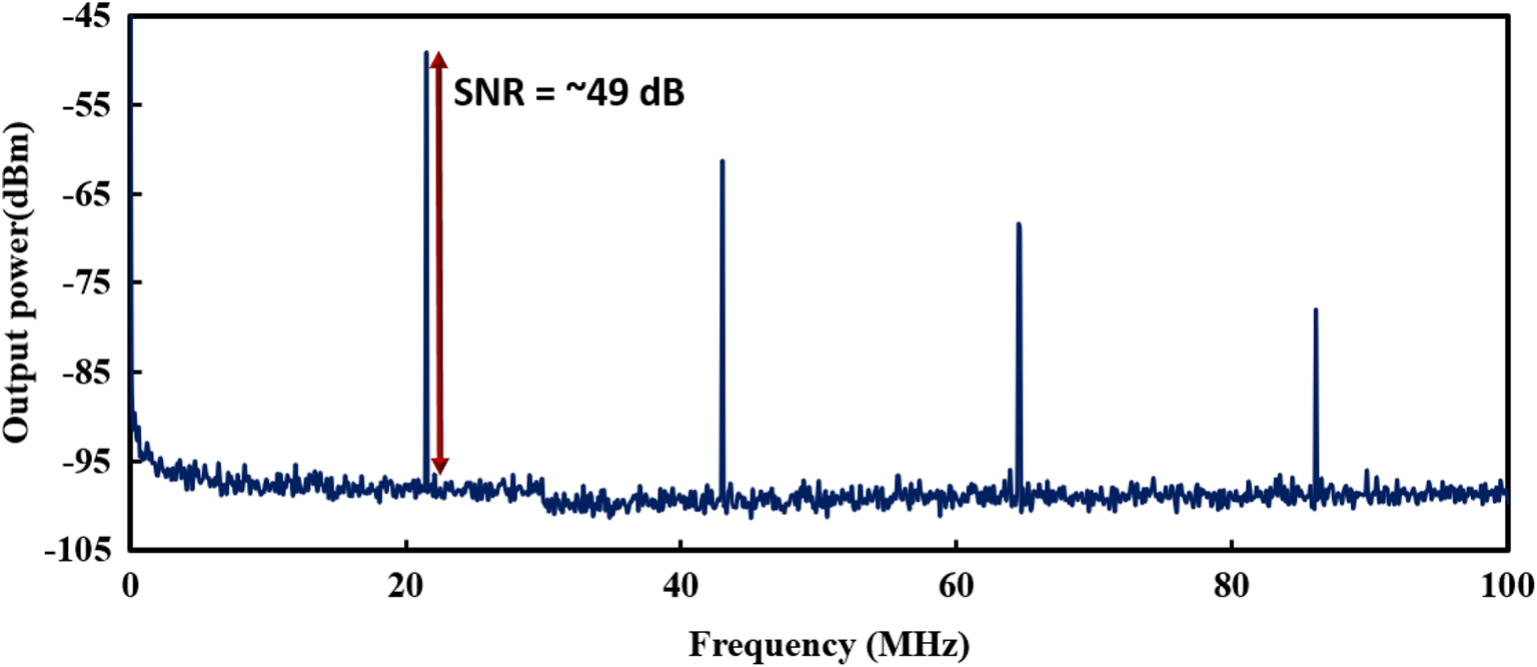
RF spectrum of DW dark pulse EDFL at pump power of 195.4 mW.

The stability of the DW dark pulse EDFL is investigated using RF spectrum analyzer. As shown in Fig. 5, the DW dark pulse EDFL is operated at fundamental frequency of 21.5 MHz, which is well-agreed with the pulse train in temporal domain. The SNR of the laser is around 49 dB, which indicates that the laser is operated in a stable condition. Even though there are two lases oscillating in the cavity, only a single frequency component is observed, which confirms a typical DW operation whereby the topological defects induced by two lases generates a single frequency component.

## Conclusion

In this work, we experimentally demonstrated the generation of domain-wall dark pulse in an Erbium-doped fiber laser using SMS fiber structure to function as interferometer and artificial saturable absorber. The SMS fiber is a combination of a 10 cm grade index multimode fiber sandwiched by single mode fibers. The interaction of dual-wavelength centered at 1567.2 nm and 1569.4 nm produces the topological defect in temporal domain and achieved a dark pulse formation with repetition rate of 21.5 MHz. The highest average pulse energy is calculated as 769.6 pJ with pulse width of 5 ns. Throughout the operating pump power range, the average pulse energy and output power increase linearly, with R^2^ of 0.9999 and achieved the laser efficiency of 9.33%. The stability of the DW dark pulse EDFL is measured in frequency domain, with SNR of 49 dB. As compared to reported DW dark pulse works, the proposed structure only required a short length of multimode fiber, which allowed the domain-wall dark pulse to achieve higher pulse repetition rate.

## Data Availability

The datasets used and/or analyzed during the current study available from the corresponding author on reasonable request.
